# Benign oral vascular lesions treated by sclerotherapy with ethanolamine oleate: A retrospective study of 43 patients

**DOI:** 10.4317/medoral.22253

**Published:** 2018-02-25

**Authors:** Diego-Tetzner Fernandes, Rogério-de Andrade Elias, Roger Santos-Silva, Pablo-Agustin Vargas, Márcio-Ajudarte Lopes

**Affiliations:** 1 DDS, MsC. Oral Diagnosis Department, Piracicaba Dental School, University of Campinas – UNICAMP, Piracicaba, São Paulo, Brazil; 2 DDS. Oral Diagnosis Department, Piracicaba Dental School, University of Campinas – UNICAMP, Piracicaba, São Paulo, Brazil; 3 DDS, PhD. Oral Diagnosis Department, Piracicaba Dental School, University of Campinas – UNICAMP, Piracicaba, São Paulo, Brazil

## Abstract

**Background:**

Although sclerotherapy is a common treatment for benign oral vascular lesions, there is no well-standardized protocol for this purpose. The aim of the present study was to describe the clinical characteristics of patients treated by sclerotherapy with ethanolamine oleate (EO), in order to contribute to a better understanding of this technique.

**Material and Methods:**

Medical records and images of 90 patients treated by the same sclerotherapy protocol were retrieved and analysed. Thus, 43 cases with complete information were selected and described.

**Results:**

The most affected age group was 41–70 years, with a female predominance and 86% of patients being Caucasian. Lips were the most affect site (70%) followed by the tongue (16%). Regarding clinical appearance, approximately 90% of lesions were classified as nodules, and 90% of patients reported no pain. Approximately 40% of lesions were 0.5–1.0 cm in size. In 58% of the patients, only one application of ethanolamine oleate was necessary. The application doses varied according to the lesion size and number of applications. Complete clinical regression occurred in 91% of cases, whereas 9% showed partial regression.

**Conclusions:**

Sclerotherapy with EO is an acceptable, effective and affordable treatment for benign oral vascular lesions.

** Key words:**Hemangioma, vascular malformations, varicose veins, sclerotherapy.

## Introduction

Hemangiomas and vascular malformations are benign lesions of blood vessels relatively common in the head and neck region. More than 60% of all hemangiomas occur in these areas, and the most affected oral regions are the lips, tongue, buccal mucosa, gums, and palate. There is a higher prevalence in females (65%), twins and preterm infants (1-3). Vascular lesions are usually asymptomatic, varies in size from a few millimetres to several centimetres, and can lead to facial asymmetries. The colour ranges from red to purple, according to the location and depth of tissue invasion, as well as the degree of vascular congestion of the affected area (4). It can present as a flat or raised lesion with a smooth or nodular surface, defined edges, sessile or pedunculated and a soft consistency on palpation.

In contrast to hemangiomas and vascular malformations, oral varices are more frequent in adults over 60 years. Age is a predisposing factor, as is the loosening of tissue and increase in venous pressure. Sublingual varices are the most common type and appear as bluish-purple single or multiple nodules in the ventral-lateral border of the tongue. Although less frequent, varices can also occur in the lips and other regions of the oral mucosa (5,6).

The diagnosis of oral vascular lesions is based on the clinical characteristics and history of the lesion. Diascopy, aspiration of the lesion and imaging exams may also contribute to diagnosis and treatment planning in some cases. In addition, important findings such as the hemodynamics of the lesion (high or low blood flow) should be considered for the treating practice (7-10).

Surgical excision is one of the most used treatments, especially for small lesions. However, before this option is selected, some problems must be considered, such as bleeding, incomplete resection and aesthetic problems (11-13). Alternative methods are mentioned in the literature, including laser surgery, cryotherapy, chemotherapy agents, corticosteroids, embolization and sclerotherapy (13-19).

The mechanism of action of sclerotherapy involves the substitution of the vascular component by a fibrotic tissue in response to an inflammatory process. Although sclerotherapy is one of the most versatile and advantageous treatments, the concentration of the sclerosing agent, dose and mode of application are not properly standardized. This applies to a wide variety of techniques and protocols, making the decision to use this treatment option extremely difficult. The development of a protocol must respect the morphological and functional uniqueness of each lesion in order to define an appropriate dosage of the sclerosing agent to be used (11).

Thus, the aim of the present study was to describe the clinical characteristics of patients with oral vascular lesions treated by sclerotherapy in a single institution, in order to contribute to a better understanding of this technique that still has no well-established protocol in the scientific literature.

## Material and Methods

Medical records and images of patients with oral vascular lesions treated by sclerotherapy were retrieved for the period between 1990 and 2010. Patient information and images were analysed and data regarding age, gender, skin colour, type and period of complaint, clinical diagnosis hypothesis, symptomatology, location, size and clinical appearance of the lesion, treatment and outcomes were collected.

The same treatment protocol with a concentration of 5% pure ethanolamine oleate (EO) (Ethamolin®, Zest Pharma Ltda., Rio de Janeiro, RJ) was applied in all lesions. The following sclerotherapy protocol was applied: a) the patients were informed regarding the treatment and probable discomfort after the procedure and their consent were obtained; b) The dose of EO was calculated according to the size of the lesion. For lesions smaller than 1.0 cm, only one application of 0.3 ml of EO was planned. For larger lesions, the size was multiplied in cm by 0.3 ml. Furthermore, for larger lesions, more sessions were often necessary and the total dose was divided among the total number of sessions; c) anaesthetic infiltration with a vasoconstrictor was performed around the lesion; d) with an insulin syringe, the EO was applied in the centre and in the deepest portion of the lesion. For larger lesions, more than one puncture point was made to distribute the sclerosing agent homogeneously. e) analgesic was prescribed for the following 2 days; f) the patients were evaluated after one week; g) the procedures were repeated at 3-week intervals until a satisfactory result was obtained.

This present study was conducted in accordance with ethical principles and was approved by the Research Ethics Committee (protocol 083/2013; 09/10/2013).

- Statistical analysis

The lesions were divided into 3 groups which values represented the average size of the lesions. The statistical analysis was done using chi-squared test through the software MedCalc (Version 11.6.1.0, Mariakerke, Belgium) with a significance level of 5%.

## Results

A total of 90 patients treated by sclerotherapy using EO were retrospectively identified. However, in 47 (52.%) of cases, detailed information regarding the doses of the applications was not total available in the medical records. Thus, 43 cases with complete information were selected and described.

The most affected age group was between 41 and 70 years (mean age: 47 years, range: 4-87 years). The female gender was predominant (56%), and 86% of patients were Caucasian. Half of the patients reported the presence of a lesion (complaint period) lasting longer than 5 years. Regarding the location, there was a higher rate of occurrence on the lips (70%) followed by the tongue (16%) and buccal mucosa (14%). According to the clinical appearance of the lesions, approximately 90% were classified as nodules. A total of 41% of the lesions were between 0.5 cm and 1.0 cm in size ( Table 1). Swelling was the most common type of complaint, reported by 28 patients (65%) followed by colour alteration (7 patients, 16%). The vast majority of patients reported no pain (90%). Concerning the clinical diagnostic hypothesis, 40 lesions were defined as hemangiomas and vascular malformations (93%) and 3 as varices (7%).

**Table 1:**
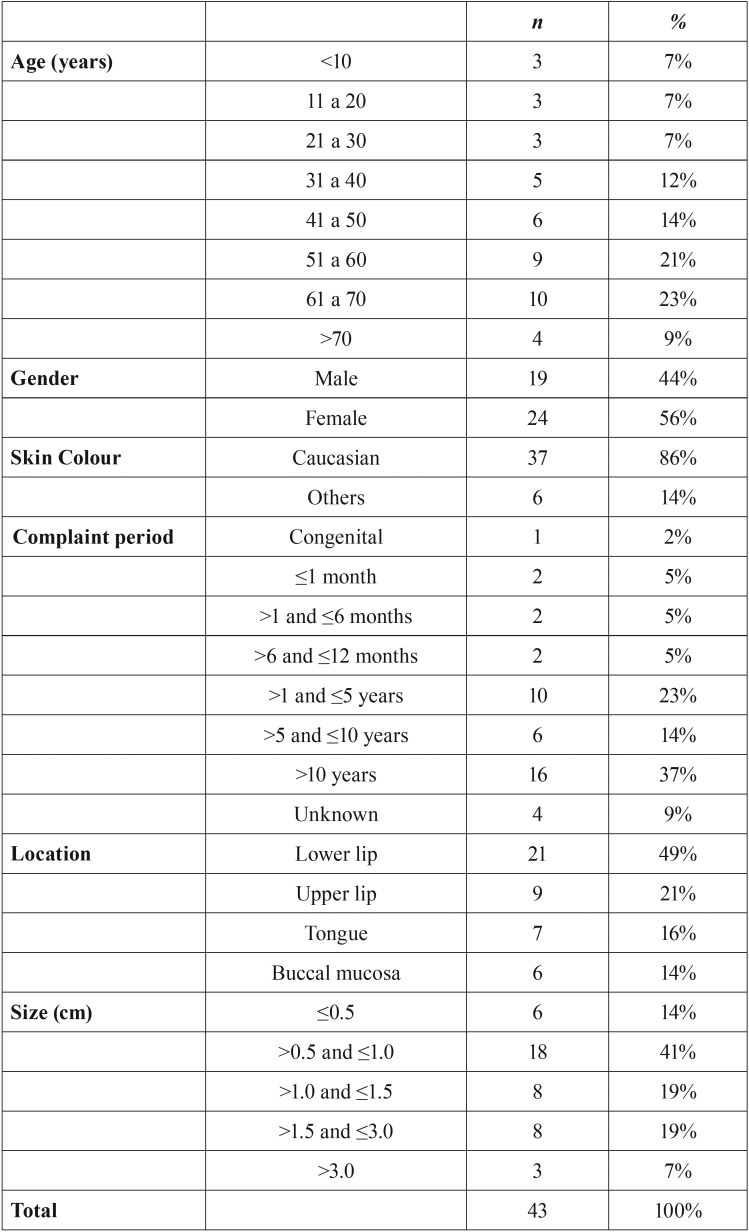
Distribution of patients treated by sclerotherapy according to age, gender, skin colour, period of complaint, location and size of the lesions.

Only one session of one application of 5% EO was sufficient to obtain a satisfactory result in 58% of the patients. The regression of the lesions usually occurred in 2 to 3 weeks after the application. Regarding the lesions larger than 1.0 cm, 6 cases received a total dose of 0.4 to 0.7ml of EO, 9 cases received 0.8 to 2.0 ml and 4 cases received more than 2.0 ml.

A positive correlation between the number of EO applications and the total doses administered was observed. Of the 20 patients who received a total dose lower than 1 ml, 75% received only one application. On the other hand, of the 23 patients who received a total dose equal or higher than 1.0 ml, 52% of these patients required 2 or more applications. A relation of the number of applications with the mean of lesion size and the mean of the total dose is shown in  Table 2. Moreover, there was a positive correlation between the total dose and the size of the lesions (*p* < 0.05) as well as between the number of applications and size of the lesions (*p* < 0.05) ( Table 3).

**Table 2:**
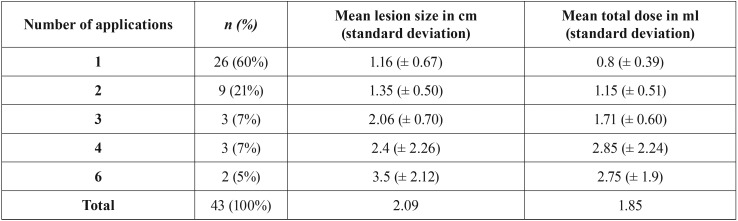
Number of cases, mean of lesion size and mean of total dose according to the number of applications.

**Table 3:**
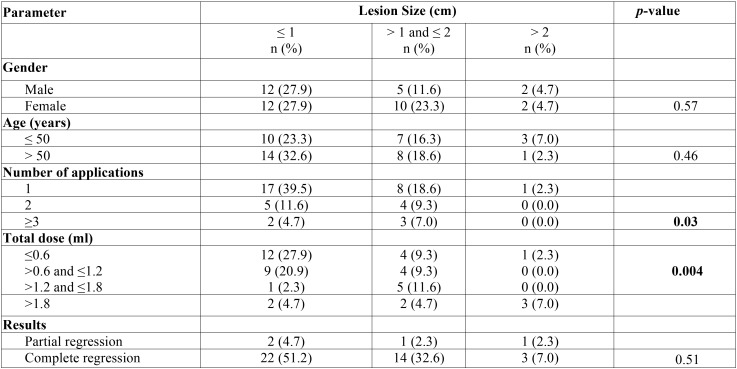
Association of clinical variables with lesion size.

Most patients reported some discomfort following application of the sclerosing agent, such as pain, swelling, redness and burning, which lasted from 1 to 3 days. However, no important complications were related or observed in any case.

**Figure 1:**
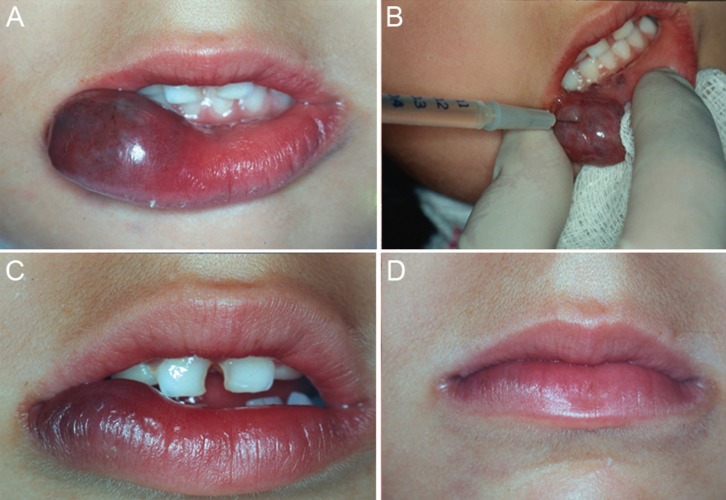
Female patient, 5 years old, with a congenital lesion in the lower lip. A. Initial clinical aspect of the lesion. B. Intralesional application of EO. C. The result after two applications. D. Final clinical aspect showing a complete regression of the lesion after six applications of EO.

**Figure 2:**
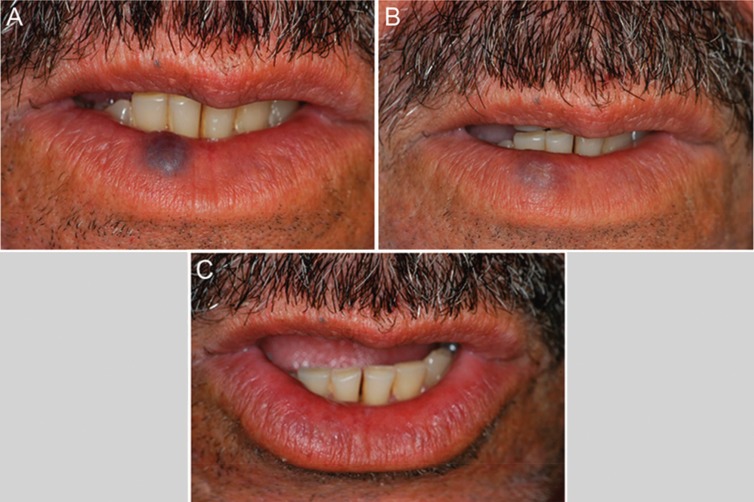
Male patient, 56 years old with a congenital lesion in the lower lip. A. Clinical aspect in the first moment. B. After 1 application of EO. C. Complete regression of the lesion after 2 applications.

**Figure 3:**
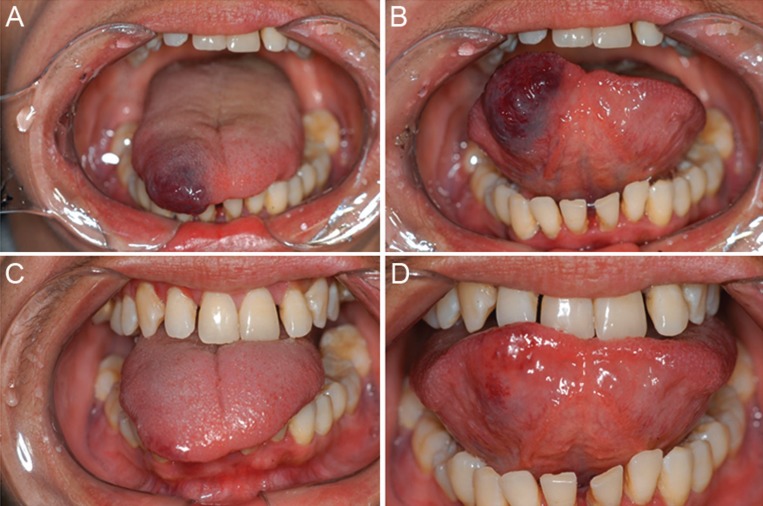
Female patient, 39 years old, with tongue lesion present for 15 years. A/B. Initial clinical aspect. C/D. Partial and functional result after two applications of EO.

The majority of lesions showed good results. Complete regression (100% of re-establishment of normal mucosa appearance) occurred in 39 patients (91%) (Figs. 1,2). Four patients (9%) showed partial regression of the lesions, and no subsequent surgery for aesthetic and/or functional rehabilitation was required (Fig. 3). No complications or recurrences were reported during the follow-up period.

## Discussion

The classification suggested by the International Society for the Study of Vascular Anomalies (ISSVA) (20) is based on the fundamental separation of vascular anomalies into those lesions with a proliferative component (named “vascular tumours”) versus relatively static “vascular malformations”, following Mulliken and Glowacki’s seminal work (1). Thus, a significant disparity between the epidemiological data is still found. In oral lesions, the similarity of the clinical characteristics of hemangiomas and vascular malformations may make difficult the classification and clinical differential diagnosis. However, the distinction of these lesions will not interfere with the proper administration of treatment in these cases (21). In the current study, according to clinical characteristics, 93% of the treated lesions were clinically classified as hemangiomas and vascular malformations and 7% were classified as varices. In contrast to the results of Corrêa *et al.* (2007) (4), varices of the ventral-lateral tongue were not included herein.

The knowledge and treatment of oral vascular lesions are of great relevance to clinicians, because some routine procedures, such as surgical interventions, could lead to significant bleeding. Sclerotherapy treatment is generally performed in cases with risk of bleeding and compromise of aesthetic and/or physiological functions (such as speech and chewing). In this context, some important characteristics should be considered, including age of the patient, size, location and hemodynamics of the lesion (high or low blood flow). The choice of treatment should be mainly based on these aspects and on the professional’s experience. In asymptomatic cases and in the absence of other disorders, clinical follow-up could be the best approach.

Sclerotherapy can provide satisfactory results in lesions of various sizes, especially in regions (face and lips) where other treatment options, such as surgery, could compromise the physiology and final aesthetic aspects. The technique contraindications involve uncontrolled diabetic patients, pregnant patients, lactating women and regions with secondary infection in which treatment could cause oedema and bleeding in the damaged area. Complications, such as exacerbated tissue necrosis and undesired triggering of an anaphylactic reaction to the drug, were related to a larger injection volume than recommended (13,19,22).

Many substances have been used for vascular lesion sclerosis, including ethanolamine oleate (EO), sodium morrhuate, sodium tetradecyl sulfate, sodium psylliate, hypertonic solution, 75% glucose, absolute alcohol, bleomycin and others (12,22,23). Studies have demonstrated the effectiveness of these solutions; however, each of them has its own characteristics, indications and side effects (11,24).

EO has been used for more than 60 years and, according to the scientific literature, causes less damage to the connective tissue. The side effects of EO are limited and can be easily avoided when it is used appropriately (11-13,23). For these reasons and due to the facility of getting this medication in Brazil, EO has been the sclerosing agent of choice in various services. The mechanism of action of EO involves an intra- and extravascular inflammatory response and fibrosis in the endothelium. The oleic acid portion causes an inflammatory reaction and also could activate coagulation, whereas the ethanolamine portion organizes the repression of the fibrin clot. Thus, the vascular lesion is replaced by fibrosis (25). These actions allow for a haemostatic balance and prevent bleeding after administration to vascular lesions (11,13,25).

Sclerotherapy may be considered a standard treatment for oral vascular lesions; however, quite different protocols were found in the scientific literature. Despite the simplicity and facility of the technique, the possibility of associated complications should not be ignored. The incidence and intensity of these complications depend directly on the technique used, the type and concentration of the sclerosing agent and the experience of the professional (11,25-27).

Regarding the concentration of EO, Johan *et al.* (11) studied two different concentrations (1.25% and 2.5%) and found no difference in the final results and post-application symptomatology. Likewise, Bonan *et al.* (25) reported that dilution of EO in distilled water resulted in no reduction of signs and symptoms. Also, dilution may have been responsible for reducing the action of the chemical agent in the vessel wall, resulting in a partial regression of lesions as described for two of the six lesions treated by these authors.

The total number of applications, the response of the lesion and the success of the treatment varies and depends on different factors, such as the protocol used and the vascular flow (hemodynamic) of the lesion. Our experience shows that low-flow lesions, soft on palpation and with a slow return of blood volume after compression, often have a better and faster response to treatment and regress with fewer applications. On the other hand, the expected effect is not always successful for lesions with high blood flow.

In the present study, for lesions smaller than 1.0 cm, 0.3 ml of EO was injected per session, often resulting in complete regression of the lesion with only one or two applications. Although 60% of cases required only one application of EO for a satisfactory result, in some lesions, more applications were necessary.

Even when higher doses are required, the intralesional application of 5% EO can be used safely when the applied dose is compatible with the size of the lesion. Thus, the occurrence of significant complications, such as necrosis of adjacent tissue or any kind of systemic alterations, is avoided. Matsumoto *et al.* (28) suggested that doses lower than 1 ml of 5% EO can provide an important factor to prevent such complications.

Furthermore, there are conflicting views with respect to the local anaesthetic application prior to the sclerosing agent injection. The use of no local anaesthesia can be justified by the maintenance of a painful sensation. This would allow the professional to stop the procedure when the sclerosing agent reached healthy tissues, thus avoiding possible undesirable damage. However, the use of anaesthetics with vasoconstrictors can increases the action time of the drug in the lesion due to peripheral vasoconstriction. Also, the anaesthetic procedure may avoid immediate pain symptoms.

Of all the 90 cases retrieved in this study, only four were treated with a combination of sclerotherapy and posterior surgery due to the size of the lesion. The surgical procedure was performed to remove the fibrous remnant of the lesion for the purpose of aesthetic and/or functional recovery. Two of these cases were previously published by our group (13).

Knowledge of the treatment options for oral vascular lesions is important for clinicians because they are relatively common in clinical practice. Although sclerotherapy is an acceptable and affordable treatment option for these lesions, several different sclerotherapy protocols were found in the scientific literature, showing variability in the concentration of the sclerosing agent, dose, and mode of application. This wide variety of techniques and protocols can difficult the choose of this particular treatment by clinicians. Herein, 43 cases of benign oral vascular lesions were treated with the same sclerotherapy protocol, which is used for more than 25 years in our institution and showed successful results.

## Conclusions

Oral vascular lesions occurred over a wide age range with a female gender predominance. Clinically, these lesions appeared mainly as an asymptomatic nodular swelling, with variable size and frequently located in the lips, buccal mucosa and tongue. Sclerotherapy with EO is an affordable and acceptable treatment option showing effectiveness when properly used.
